# FABP6 serves as a new therapeutic target in esophageal tumor

**DOI:** 10.18632/aging.205448

**Published:** 2024-01-25

**Authors:** Dengfeng Zhang, Fangchao Zhao, Haitao Liu, Pengfei Guo, Zhirong Li, Shujun Li

**Affiliations:** 1 Department of Thoracic Surgery, The Second Hospital of Hebei Medical University, Shijiazhuang, Hebei 050000, China; 2 College of Life Science, Inner Mongolia University, Hohhot, Inner Mongolia 010031, China

**Keywords:** esophageal tumor, FABP6, Treg, prognostic biomarker, migration

## Abstract

Background: Esophageal cancer is one of the most common malignant tumors with high incidence and mortality rates. Despite the continuous development of treatment options, the prognosis for esophageal cancer patients remains poor. Therefore, there is an urgent need for new diagnostic and therapeutic targets in clinical practice to improve the survival of patients with esophageal cancer.

Methods: In this study, we conducted a comprehensive scRNA-seq analysis of the tumor microenvironment in primary esophageal tumors to elucidate cell composition and heterogeneity. Using Seurat, we identified eight clusters, encompassing non-immune cells (fibroblasts, myofibroblasts, endothelial cells, and epithelial cells) and immunocytes (myeloid-derived cells, T cells, B cells, and plasma cells). Compared to normal tissues, tumors exhibited an increased proportion of epithelial cells and alterations in immune cell infiltration. Analysis of epithelial cells revealed a cluster (cluster 0) with a high differentiation score and early distribution, suggesting its importance as a precursor cell.

Results: Cluster 0 was characterized by high expression of FABP6, indicating a potential role in fatty acid metabolism and tumor growth. T cell analysis revealed shifts in the balance between Treg and CD8+ effector T cells in tumor tissues. Cellular communication analysis identified increased interactions between FABP6+ tumor cells and T cells, with the involvement of the MIF-related pathway and the CD74-CD44 interaction. This study provides insights into the cellular landscape and immune interactions within esophageal tumors, contributing to a better understanding of tumor heterogeneity and potential therapeutic targets.

## INTRODUCTION

Esophageal cancer is among the most prevalent malignancies worldwide, with high incidence and mortality rates. In recent years, it has shown an increasing trend and tends to affect younger individuals. It ranks as the eighth most common cancer and the sixth leading cause of cancer-related death globally [1, 2]. Despite the continuous development of treatment modalities, such as surgery combined with radiotherapy and chemotherapy, the prognosis for patients with esophageal cancer remains generally poor. In most countries, the 5-year survival rate after diagnosis is still low, ranging from 10% to 30% [3]. The inactivation of tumor suppressor genes and activation of oncogenes play crucial roles in the development of esophageal cancer [4, 5]. Therefore, there is an urgent need for novel diagnostic and therapeutic targets in clinical practice to enhance the survival of patients with esophageal cancer.

Single-cell technology is an area of research and analysis of individual cells that utilizes high-throughput sequencing and other techniques to reveal information on gene expression, genomic variations, protein expression, and other aspects of a single cell [6]. In this article, we conducted a comprehensive scRNA-seq analysis of the tumor microenvironment in primary esophageal tumors to elucidate cell composition and heterogeneity. Using Seurat, we identified eight clusters, encompassing non-immune cells (fibroblasts, myofibroblasts, endothelial cells, and epithelial cells) and immunocytes (myeloid-derived cells, T cells, B cells, and plasma cells). Compared to normal tissues, tumors exhibited an increased proportion of epithelial cells and alterations in immune cell infiltration. Analysis of epithelial cells revealed a cluster (cluster 0) with a high differentiation score and early distribution, suggesting its importance as a precursor cell. Cluster 0 was characterized by high expression of FABP6, indicating a potential role in fatty acid metabolism and tumor growth. T cell analysis revealed shifts in the balance between Treg and CD8+ effector T cells in tumor tissues. Cellular communication analysis identified increased interactions between FABP6+ tumor cells and T cells, with the involvement of the MIF-related pathway and the CD74-CD44 interaction. This study provides insights into the cellular landscape and immune interactions within esophageal tumors, contributing to a better understanding of tumor heterogeneity and potential therapeutic targets.

## MATERIALS AND METHODS

### Data availability

Retrieve the single-cell dataset pertaining to Esophageal Squamous Cell Carcinoma (ESCC) from Gene Expression Omnibus (GEO; https://www.ncbi.nlm.nih.gov/geo/) with dataset ID GSE160269. The dataset originates from Homo sapiens and its data platform is GPL24676. For transcriptome data, we downloaded the TCGA-ESCA dataset from the TCGA GDC website (https://portal.gdc.cancer.gov/). In addition, we supplemented the control group with normal tissue information obtained from the GTEx Portal website (https://www.gtexportal.org/home/). The FPKM normalized gene expression data and clinical information for ESCC were obtained from The Cancer Genome Atlas (TCGA). In R, the “surv_cutpoint” function from the survminer package was employed with a median value (Cutoff-High (%) = 50, Cutoff-Low (%) = 50) as the cutoff point for survival analysis. Kaplan-Meier method was utilized to measure survival curves, and *p*-values were calculated using the log-rank test.

### Single-cell RNA-seq analysis

The R package Seurat [7] was employed to identify distinct cell types and investigate variations in immune cell infiltration. To ensure data quality, cells were filtered based on the following criteria: those with fewer than 100 genes, over 7500 genes, or more than 4% mitochondrial expression were excluded from the analysis. Normalization of raw counts was performed using the “NormalizeData” function in Seurat. Next, the “FindVariableGenes” function was applied to identify genes with significant variation across the dataset. To prepare the data for dimensionality reduction, the “ScaleData” function in Seurat was used to scale and center the expression values. For dimensionality reduction, Principal Component Analysis (PCA) and Uniform Manifold Approximation and Projection (UMAP) were implemented. The first 20 dimensions resulting from PCA and UMAP were selected for visualization purposes. To cluster the cells based on their gene expression profiles, the “FindClusters” function in Seurat was employed. This allowed for the identification of distinct cell populations within the dataset. To determine highly expressed genes specific to each cell cluster, the “FindAllMarkers” function was utilized. This function helps identify genes that are significantly enriched in specific cell populations. Furthermore, to identify differentially expressed genes (DEGs) between two cell populations, the “FindMarkers” function was applied. This function facilitates the identification of genes that show significant expression differences between the specified cell populations. Overall, the combination of these Seurat functions provided a comprehensive analysis pipeline for cell type identification, exploration of immune cell infiltration patterns, and identification of differentially expressed genes in the dataset.

### Cell-cell interaction analysis

CellChat [8] was employed to predict potential cell-cell interactions. The raw counts and cell type annotations of each cell were inputted into CellChat for analysis. This software enables the modeling of intercellular communication through three core modules: (1) cross-referencing the ligand-receptor interaction database to identify potential cell-cell signaling relationships, (2) utilizing network algorithms to infer and visually map communication pathways between cell types, and (3) leveraging quantitative techniques to analyze the dynamics and relative strengths of intercellular signaling interactions. Together, these three pillars provide an integrated platform for examining and characterizing the complex signaling networks underlying multicellular system behavior.

### Pseudo-time analysis

To explore the developmental timing and trajectory of esophageal tumor cells, we employed Monocle [9]. This powerful tool applies advanced algorithms to infer the sequential changes in gene expression that each cell undergoes as part of a dynamic biological process. By utilizing unsupervised or semi-supervised learning, Monocle constructs a trajectory of cells arranged in a simulated time sequence based on the input data. This enables us to gain insights into the developmental progression of esophageal tumor cells and understand the underlying molecular changes driving their differentiation.

### Cell-cell ligand receptor interaction analysis

We employed NicheNet [10] to predict the ligands responsible for driving transcriptome changes in target cells. This computational approach utilizes the expression profiles of both sender and target cells to construct potential ligand-receptor interactions. What sets NicheNet apart is its innovative ability to predict the ligand-target gene connections between interacting cells by integrating cell expression data, signal intensity, and gene regulation networks. By leveraging these factors, NicheNet enables the identification of crucial ligands involved in cell communication, shedding light on the molecular mechanisms underlying cellular interactions and their impact on gene expression patterns.

### Gene enrichment analysis

The gene enrichment analysis of the identified genes obtained through the “FindAllMarkers” function was performed using the R package Cluster Profiler [11]. Cluster Profiler is a widely utilized R package that offers a comprehensive set of tools for interpreting omics data. It enables functional annotation and enrichment analysis of diverse gene sets, along with the visualization of enrichment analysis results. With its support for multiple gene sets and pathway databases, Cluster Profiler proves to be an excellent choice for our study, providing valuable insights into the functional significance and biological pathways associated with the identified genes.

### Assessing the heterogeneity of single-cell populations

In our study, we employed ROGUE [12], a universal entropy-based metric, to assess the purity of the major cell populations and compare their heterogeneity. The ROGUE algorithm calculates the purity of a specific cell cluster, yielding an index that falls within the range of zero to one. A higher index value indicates a higher level of purity within the cluster. A score of zero corresponds to the most heterogeneous cell population, while a score of one signifies a completely pure population. By utilizing ROGUE, we were able to quantitatively evaluate the homogeneity of the cell populations and gain insights into the degree of cellular diversity within our study.

### Pathway enrichment analysis

In our analysis, we employed PROGENy [13] to predict the activity of 14 signaling pathways, including androgen, estrogen, EGFR, hypoxia, JAK-STAT, MAPK, NFK-B, PI3K, p53, TGF-b, TNF-a, Trail, VEGF, and WNT, using gene expression data. PROGENy assigns a weight to each gene based on its responsiveness to the corresponding pathway disturbance, indicating the magnitude and direction of pathway adjustment. Subsequently, the pathway score is calculated as the weighted sum of the product of gene expression and weight. PROGENy is widely utilized as a tool for inferring pathway activity from gene expression data. It provides valuable insights into the biological processes that are altered in a given sample, offering a deeper understanding of the signaling pathway dynamics at play. By leveraging PROGENy, we were able to gain insights into the activity levels of these specific pathways and unravel their potential roles in the biological context under investigation.

### Transcription factor activity analysis

To analyze transcription factor activity in single cells, we utilized the SCENIC algorithm, which was first introduced in the prestigious scientific journal Nature Methods in 2017 [14]. SCENIC employs co-expression and motif analysis techniques to reconstruct gene regulatory networks based on single-cell transcriptome data. By leveraging the information contained within the cis-regulatory network, SCENIC enables the identification of transcription factors and cell states. One of the key advantages of SCENIC is its ability to automatically eliminate batch effects, such as tumor sample specificity, by focusing on biologically driven features. This ensures that the analysis is robust and accurately captures the underlying regulatory dynamics within the single-cell transcriptome data. By utilizing SCENIC, we were able to gain insights into the activity levels of transcription factors and understand their roles in driving cellular heterogeneity and regulatory processes within the studied context.

### Patient samples

The tumor tissues and matched adjacent normal esophageal tissues were collected from six patients undergoing surgical resection at the Second Hospital of Hebei Medical University in July of 2023. All patients provided written informed consent, and the study was approved by the Research Ethics Committee of the Second Hospital of Hebei Medical University.

### Cell lines

The esophageal cancer cell lines KYSE30, KYSE150, ECA109, and normal esophageal epithelial cell line HET1A were purchased from Procell Life Science & Technology Co., Ltd (Wuhan, China). All cell lines were cultured according to the manufacturer's instructions.

### Detection of mRNA expression by quantitative fluorescence polymerase chain reaction (qRT-PCR)

RNA was extracted from the samples using TRIzol reagent (Invitrogen, Grand Island, NY, USA), a commercially available reagent used for isolating total RNA from cells and tissues for PCR analysis. The extracted RNA was subjected to reverse transcription using Superscript III Transcriptase (Invitrogen), following the manufacturer’s instructions. Quantitative real-time PCR (qRT-PCR) was performed using SYBR green and a Bio-Rad CFX96 system to quantify the mRNA expression level of the target genes. The initial step involved incubating the samples at 50°C for 2 minutes, followed by heating to 95°C for 8 minutes and 30 seconds. This was followed by 45 cycles at 95°C for 15 seconds each, and 60°C for 1 minute. The extension step included 95°C for 1 min, 55°C for 1 min, and 55°C for 10 s. The expression levels were normalized to the expression of GAPDH, which served as the internal control. The primer sequences used for qRT-PCR are listed below:

FABP6 5′-ACCGGCAAGTTCGAGATGG-3′
FABP6 3′-CCTTTTCGATTACATCGCTGGA-5′
GAPDH 5′-TGTGGGCATCAATGGATTTGG-3′
GAPDH 3′-ACACCATGTATTCCGGGTCAAT-5′.

### siRNAs transfection

To downregulate FABP6 expression, siRNA-FABP6#1 and siRNA-FABP6#2 (GenePharma) were employed, while a non-specific siRNA-NC (GenePharma) served as a control. The Lipofectamine RNAiMAX was used to facilitate siRNA transfection, following the manufacturer’s instructions. After a 48-hour incubation period with siRNA complexes, ECA109 and KYSE30 cell lines proceeded to the subsequent step. The sequences of the involved siRNAs are provided below:

siRNA-FABP6#1
  Sense: 5′-GGAGAGUGAGAAGAAUUAUTT-3′
  Antisense: 5′-AUAAUUCUUCUCACUCUCCTT-3′;
siRNA-FABP6#2
  Sense: 5′-GCCCGCAACUUCAAGAUCGTT-3′
  Antisense: 5′-CGAUCUUGAAGUUGCGGGCTT-3′;
siRNA-NC
  Sense: 5′-UUCUCCGAACGUGUCACGUTT-3′
  Antisense: 5′-ACGUGACACGUUCGGAGAATT-3′.

### Western blotting assay

Total proteins were extracted using RIPA whole-cell lysis solution. The proteins were separated through 8-12% SDS-PAGE electrophoresis, measured, and semi-dry transferred to PVDF membranes (Millipore, Billerica, MA, USA). The PVDF membranes were blocked with TBS + Tween (TBST) solution containing 5% skim milk powder for 2 hours, washed, and then incubated with a primary antibody (FABP6, 13781-1-AP, Proteintech, 1:50) overnight at 4°C. The membranes were subsequently washed and exposed to a secondary antibody labeled with horseradish peroxidase for 2 hours. Following the washing of the PVDF membrane, the chemiluminescent substrate was applied, and the grayscale values were measured using a gel imaging system.

### Transwell migration experiments

The cells were harvested using serum-free media and seeded into the upper chambers of polycarbonate membrane filters with a pore size of 8.0 μm (Corning Incorporated, Corning, NY, USA) at a density of 1 × 105/mL. Subsequently, 600 μL of media containing 10% FBS was added to the lower chambers, and the cells were incubated at 37°C for 36 hours. To minimize potential bias in the results, three replicate wells were established for each experimental group, and each experiment was repeated thrice. The field of view for cell counting was randomly selected, and images were concurrently captured. Using Image J software, the number of migrating cells was quantified.

### Wound-healing assay to detect cell migration

Before the addition of tumor cells, three parallel lines were marked on the back of a 6-well plate. The cells were seeded at a density of 1 × 105 cells per well and subsequently transfected with si-FABP6 and si-NC after complete adherence to the well. After 24 hours, tumor cell growth was monitored, and a 10 μL gun was used to create a scratch perpendicular to the horizontal line on the back of the 6-well plate. The cells were washed with phosphate buffer solution (PBS) and cultured in serum-free medium at 37°C with 5% CO_2_ in an incubator. The width of the scratch was measured using Image J software, and the cell migration rate was subsequently calculated. The culture medium was maintained at 37°C with 5% CO2 in an incubator for the duration of the experiment.

### Immunohistochemical stainings and evaluation

Immunohistochemical staining was conducted using 4 μm paraffin-embedded tissue cross sections. The sections were deparaffinized with xylene and rehydrated, followed by pre-incubation with 10% normal goat serum (710027, KPL, USA). Thereafter, the sections were incubated overnight at 4°C with a primary antibody (FABP6, 13781-1-AP, Proteintech, 1:50). Following washing, horseradish peroxidase-labeled rabbit IgG antibody (021516, KPL, USA) was used as the secondary antibody. The color development reaction was performed using the DAB substrate kit. Finally, the sections were counterstained with hematoxylin and evaluated after dehydration and clearance.

### Statistical methods

R software (version 4.2.1) and GraphPad Prism 8.0 were employed for statistical analysis and graphical visualization of the data. The Pearson or Spearman coefficients were calculated to determine the correlation between variables. A *P* value less than 0.05 was considered statistically significant for all statistical calculations.

### Availability of data and materials

The original datasets analyzed in this study are publicly available through the following repositories: The Cancer Genome Atlas (https://tcga-data.nci.nih.gov/), Genotype-Tissue Expression project (https://www.gtexportal.org/home/index.html), and NCBI Gene Expression Omnibus (https://www.ncbi.nlm.nih.gov/gds/). Further inquiries can be directed to the corresponding authors.

## RESULTS

### scRNA-seq analysis of the tumor microenvironment

**Figure 1:**
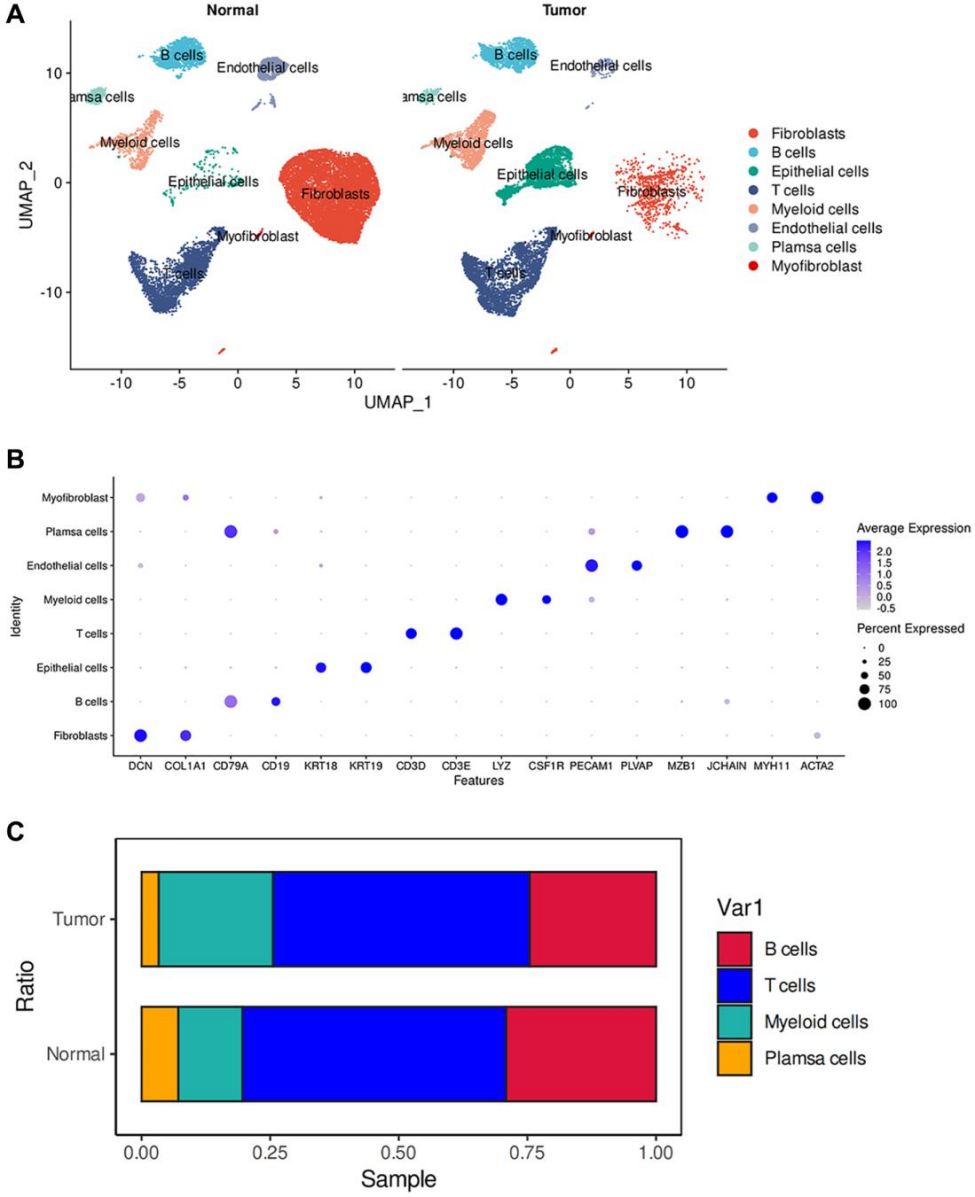
**ScRNA-seq analysis of the tumor microenvironment in esophageal cancer cells.** (**A**) The UMAP plot displays the cellular composition of the esophageal tumor microenvironment. (**B**) Dotplot showing marker gene expression in indicated cell types. (**C**) Histograms indicating the proportion of cells in tumor tissue and normal tissue.

We constructed a comprehensive tumor ecosystem map for esophageal tumor using Seurat, facilitating cell classification and identification of marker genes. The visualization of the results was accomplished through the application of the Uniform Manifold Approximation and Projection (UMAP) method, revealing 8 distinct clusters (Figure 1A). We observed non-immune cells in the clusters, primarily fibroblasts (DCN and COL1A1), myofibroblasts (MYH11 and ACTA2), endothelial cells (PECAM1 and PLVAP) and epithelial cells (KRT19 and KRT18). The immunocytes consisted of myeloid-derived cells (LYZ and CSF1R), T cells (CD3D and CD3E), B cells (CD19 and CD79A), and plasma cells (JCHAIN and MZB1) (Figure 1B). These cells are present in the esophagus of both normal and tumor patients. We observed substantial heterogeneity in the composition of cell types in normal and tumors. Among non-immune cells, tumors have more epithelial cells than normal, which may be because the cellular composition in tumors is mainly epithelioid tumor cells. Relatively, normal has more fibroblast and endothelial cell composition (Figure 1A). This suggests that in the tumor microenvironment, non-immune cells may have a key influence on tumor growth and development. Previous studies have shown that activating specific tumor-associated genes in non-immune healthy cells surrounding the tumor can induce non-apoptotic programed cell death in tumor cells [15].

We then examined the infiltration of immune cells and found no significant difference in the infiltration of T cells and B cells between normal and tumor tissues. However, myeloid cells showed a significant increase in tumors, while plasma cells exhibited a significant decrease (Figure 1C). Tumors possess a complex ecosystem in which heterogeneous malignant cells interact with immune and non-immune cells, shaping a complex cellular network in the tumor microenvironment (TME) [16]. Myeloid cells are an important component of the immune cells infiltrating tumors and play a crucial role in regulating tumor inflammation and angiogenesis [17, 18]. Persistent inflammation, such as that which occurs in cancer, disrupts normal bone marrow cell production, leading to the generation of immunosuppressive bone marrow-derived cells, such as myeloid-derived suppressor cells (MDSCs) and tumor-associated macrophages (TAMs). CD38-expressing bone marrow-derived suppressor cells promote tumor growth in an esophageal cancer mouse model [19, 20]. Therefore, an increase in infiltration of myeloid cells may indicate poor prognosis in esophageal cancer. In the tumor microenvironment, tumor-infiltrating B cells (TIL-B) can be identified based on their expression of CD19 or CD20. Several studies have correlated the effect of TIL-B on patient outcomes, revealing a heterogeneous effect based on specific tumor anatomic site, histology, and molecular subgroup [21–25]. In many clinical and human studies, high expression of B-cell markers has been associated with significantly improved outcomes [26–28]. Therefore, the absence of plasma cells may suggest a poor prognosis in esophageal cancer.

### Heterogeneity of esophageal tumor cells

**Figure 2:**
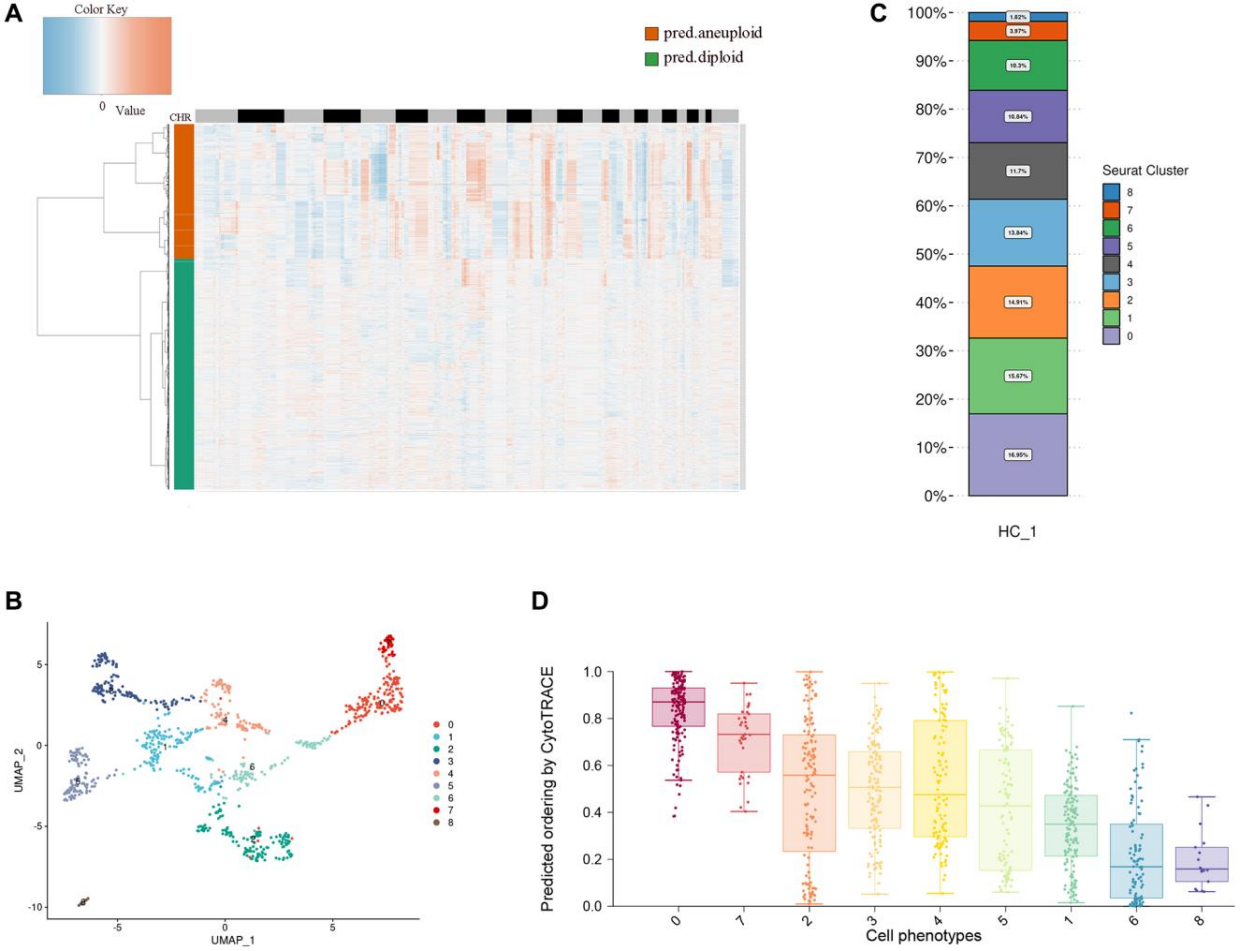
**Heterogeneity of esophageal tumor cells.** (**A**) Heatmap showing the differences in chromosomal copy numbers within the epithelial cells. (**B**) UMAP plot showing the subtypes of tumor cells. (**C**) Histograms indicating the proportions of each tumor subgroup. (**D**) Boxplot showing cellular differentiation potential of each tumor subgroup.

Esophageal tumor cells are epithelioid malignant cells. It is difficult to separate epithelial cells from malignant cells by traditional markers. Here, we use copyKAT to analyze the chromosome copy number variation (CNV) of epithelial cells and divide epithelial cells into diploid and aneuploid, through the heat map of chromosome copy number, we found that aneuploid has more chromosomal variation, so we defined polyploidy as esophageal tumor cells (Figure 2A). Research has shown that most CNVs are closely associated with complex diseases. If CNVs occur within or near tumor-associated gene sequences, they can activate oncogenes, suppress tumor suppressor genes, and ultimately contribute to tumor development [29, 30]. CNVs affect gene expression, phenotype differences, and phenotypic adaptation by altering gene dosage and regulating gene activity, leading to the development of tumors and other genetic diseases [31–35].

We performed dimensionality reduction clustering of esophageal tumor epithelial cells using unsupervised clustering and visualized nine distinct clusters with the unified manifold approximation and projection (UMAP) method. We found that the distribution of tumor cells across UMAPs is continuous, suggesting that these subpopulations may have continuity across the developmental lineage of tumor cells (Figure 2B). Through the distribution ratio of each subgroup, we found that cluster 0 has the most proportional distribution, which suggests that cluster 0 may have an important position in esophageal tumor cells (Figure 2C). Then, we predicted the relative differentiation state of cells by cytoTRACE for each subcluster, cluster 0 had the highest differentiation prediction score, and the results indicated that cluster 0 might be the starting point of all cells in the cell developmental trajectory (Figure 2D).

### The developmental trajectory of esophageal tumor cells

**Figure 3:**
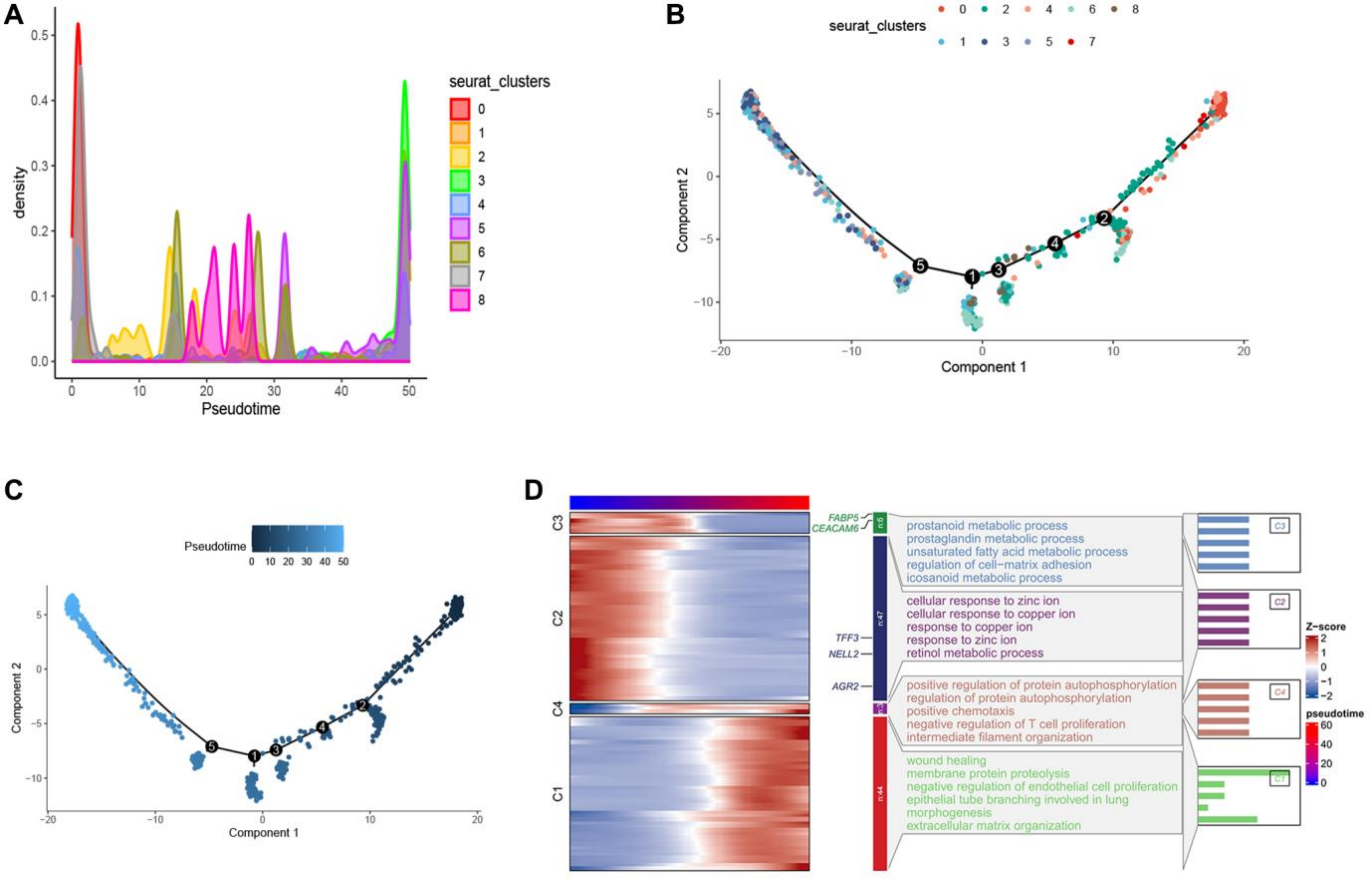
**Pseudo-time series analysis of esophageal cancer cells.** (**A**) Density map provides a visual representation of the distribution of tumor cell subtypes across a pseudo-time series. (**B**, **C**) Pseudo-time-ordered analysis of tumor cells. Tumor cell subtypes are labeled by colors. (**D**) Heatmap showing the dynamic changes in the expression of developmental genes over pseudo-time.

In order to better explore the developmental trajectory of esophageal tumor cells, we performed a pseudo-time analysis of esophageal tumor cells using Monocle2. We visualized the distribution of each cell cluster in pseudo-time via density plots and found that cluster 0 was predominantly distributed in the early stages of pseudo-time (Figure 3A). The branching tree showed that these cell clusters did not branch on the developmental lineage, suggesting that these cell clusters had developmental continuity, which was consistent with our analysis (Figure 3B, 3C).

Furthermore, we conducted Gene Ontology (GO) analysis on the pseudo-temporal genes. The results indicate that in the early pseudo-temporal stage, metabolic pathways such as unsaturated fatty acid metabolic process and icosanoid metabolic process are enriched, suggesting that cluster 0 exhibits higher metabolic activity. The enrichment of pathways like negative regulation of T cell proliferation in the late pseudo-temporal stage suggests that tumor development and maturation are associated with an immunosuppressive effect involving the inhibition of T cell differentiation (Figure 3D). FABP5, a fatty acid binding protein, plays a role in fatty acid uptake, transport, and metabolism, suggesting that cluster 0 distributed in the early pseudo-time may have higher fatty acid metabolism to generate energy for proliferation and development. The role of altered fatty acid metabolism in cancer has garnered renewed interest due to their dual roles as structural components of the membrane matrix, important secondary messengers, and fuel sources for energy production [36–39]. Experimental evidence suggests that fatty acid metabolism has profound effects on the cancer epigenome, which in turn regulates gene expression and cellular differentiation [40–42]. According to relevant literature reports, FABP5 can reprogram the fatty acid metabolism of tumors to promote tumor growth [43–47], although there is limited research on FABP5 in esophageal cancer. This suggests that FABP5 may serve as a potential diagnostic marker and therapeutic target. CEACAM6 belongs to the carcinoembryonic antigen gene family [48, 49], and increasing evidence shows that CEACAM6 participates in multiple aspects of tumor development, including promoting tumor invasion and metastasis, inhibiting tumor cell apoptosis, promoting tumor angiogenesis, and suppressing tumor cell adhesion [50–52]. AGR2 is expressed in various solid tumors [53–56]. AGR2 can affect cell signaling and metabolism by upregulating the expression of CCAAT-enhancer-binding protein β and the transcription factor hypoxia-inducible factor 2α subunit, thereby promoting tumor development [53]. Similarly, multiple studies have shown that TFF3 [57–59] and NELL2 [60] are significantly upregulated in tumor tissue compared to adjacent normal tissue, promoting tumor migration, growth, and leading to poor prognosis. These findings suggest that FABP5, CEACAM6, AGR2, TFF3, and NELL2 are highly expressed in cluster 0 and promote tumor development. The results of the appeal show that cluster 0 may be the precursor cells of these cell clusters, and store energy through higher fatty acid metabolism, providing an energy basis for subsequent differentiation, proliferation, migration and survival, which reflects the importance of cluster 0, so we will focus on the 0 cluster later.

### FABP6+ tumor cells play an important role in the progression of esophageal tumor

**Figure 4:**
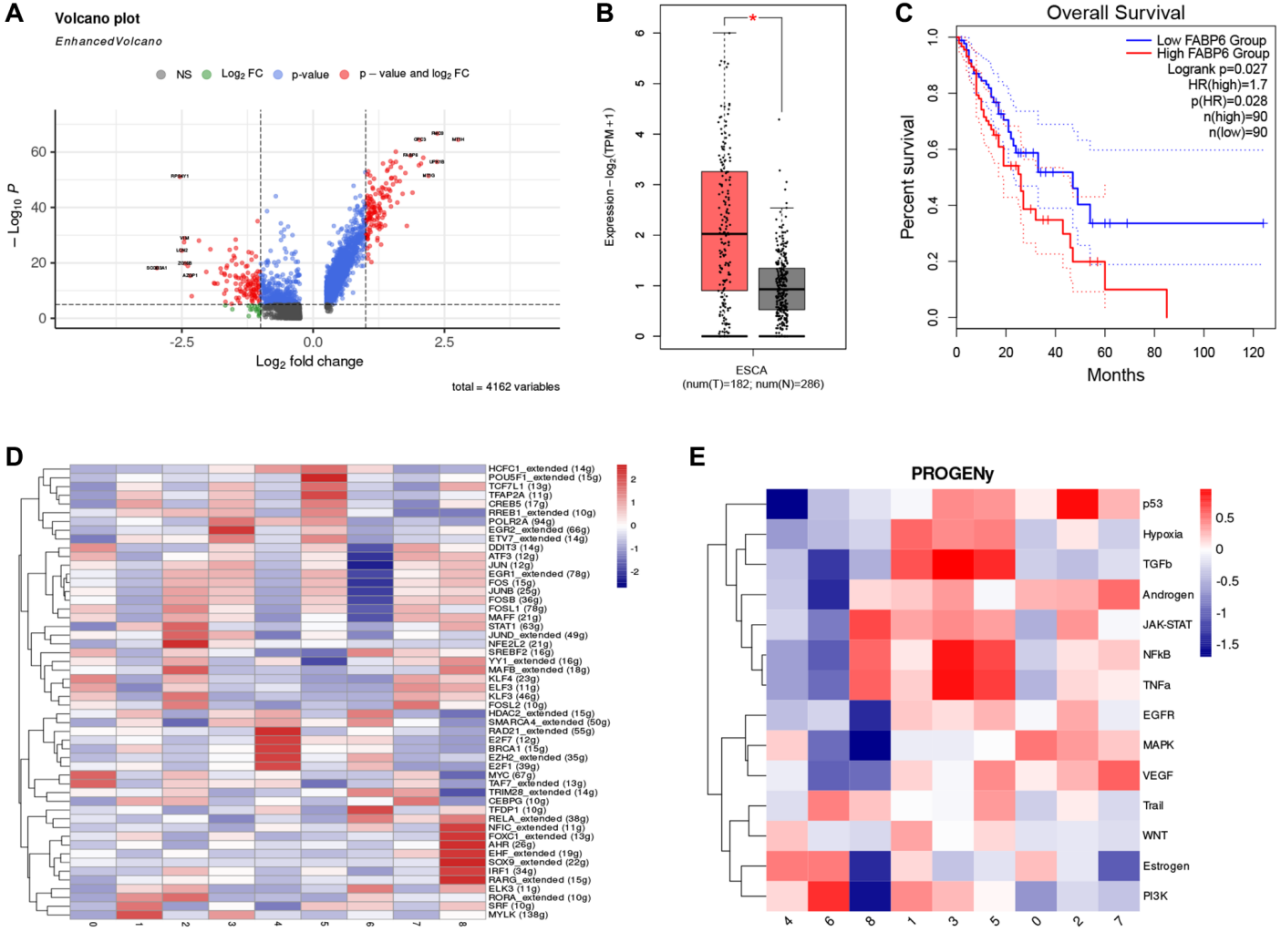
**FABP6 plays an important role in esophageal cancer.** (**A**) Volcano plot illustrates the differentially expressed genes between Cluster 0 and normal epithelial cells. (**B**) Boxplot displays the expression levels of FABP6 in esophageal cancer and normal esophageal tissues within the TCGA and GTEx database. (**C**) Survival analysis of FABP6 in the TCGA database. (**D**) Heatmap showing tumor cell transcription factor activity. (**E**) Heatmap showing the enrichment of 14 tumor-associated signaling pathways.

To further explore the gene signature of cluster 0, we visualized differential genes between normal epithelial cells and cluster 0. Results showed that compared to normal epithelial cells, cluster 0 highly expressed FABP6, which has similar functions to FABP5, including the uptake, transport, and metabolism of fatty acids (Figure 4A). We analyzed the expression of FABP6 in normal esophageal tissue and esophageal tumor tissue through the TCGA and GTEx database and found that esophageal tumor tissue had a higher expression level (Figure 4B). Further survival analysis of FABP6 revealed that patients with high expression of FABP6 had poorer survival (Figure 4C). To explore the regulatory mechanism of cluster 0, we analyzed the activity of transcription factors and found that MYC and TAF7 were significantly enriched in cluster 0 (Figure 4D). These findings suggest that FABP6 may play a critical role in the development of esophageal tumors and that MYC and TAF7 may be involved in the regulatory mechanism of cluster 0. The MYC oncogene is a member of a superfamily of genes whose products are frequently activated in human cancers [61–63]. MYC is a master regulator of multiple biological programs and primarily functions as a transcription factor that regulates the expression of thousands of genes, either directly or indirectly [64, 65]. Reports suggest that MYC overexpression can cause tumorigenesis, contributing to many of the hallmarks of cancer, including proliferation, self-renewal, cell survival, genomic instability, metabolism, invasiveness, angiogenesis, and remodeling of the tumor microenvironment [66–70]. These findings suggest that MYC may play a key role in the development and progression of esophageal tumors and may be involved in the regulatory mechanism of cluster 0. TAF7, short for TATA box-binding protein associated factor 7, is a key protein involved in transcriptional regulation processes [71]. TAF7 may play an important role in cancer, as evidenced by a study of breast cancer that found significantly increased expression levels of TAF7 in tumor tissue, which was correlated with the invasive and metastatic potential of breast cancer [72]. Additionally, other studies have found abnormal expression of TAF7 in various types of cancer, including gliomas and lung adenocarcinomas [73–75]. These findings suggest that TAF7 may be involved in the development and progression of esophageal tumors and may be a potential therapeutic target.

In addition, we performed pathway enrichment analysis on cluster 0 using PROGENy to further explore the activated signaling pathways of cluster 0 (Figure 4E). Our study found that the MAPK pathway was significantly enriched in cluster 0. MAPK pathways are cascades of three kinases, where the most upstream kinase (MAPKKK) responds to various extra- and intracellular signals and activates the middle kinase (MAPKK) by direct phosphorylation. MAPKKs exclusively phosphorylate and activate a MAPK, which typically has many substrates that execute specific cell fate decisions appropriate to the input signal [76]. Over 85% of cancers exhibit hyperactive MAPK signaling, which is directly caused by genetic alterations of its upstream activators or components, including RTKs, Ras, and BRAF, or indirectly by those independent of Ras or RAF [77–79]. This hyperactivity significantly promotes disease progression [80]. These findings suggest that the MAPK pathway may play a critical role in the development and progression of esophageal tumors, and targeting this pathway may be a potential therapeutic strategy for cluster 0. After conducting these analyses, we have defined cluster 0 as FABP6+ tumor cells. This designation is based on the observed expression of FABP6 in this particular cluster. By identifying FABP6 as a defining marker, we can distinguish and categorize cluster 0 as a specific subset of tumor cells within the tumor microenvironment. This characterization provides valuable insights into the heterogeneity of tumor cells and may contribute to a deeper understanding of their functional roles and potential therapeutic implications.

### Infiltration of T cells in esophageal tumor

T cells play a crucial role in the tumor microenvironment, and different subsets of T cells perform distinct functions. T cells can be divided into helper T cells (CD4+ T cells) and cytotoxic T cells (CD8+ T cells). Helper T cells can be further classified into subgroups such as Th1, Th2, Th17, and Treg, all of which play important roles in immune regulation [81–86]. Th1 cells, characterized by the expression of interferon (IFN)-γ lineage cytokine and the master transcription factor T-bet, participate in type 1 immune responses to intracellular pathogens such as mycobacterial species and viruses [82]. Th2 cells, characterized by the expression of interleukin (IL)-4/IL-5/IL-13 lineage cytokines and the master transcription factor GAΤA3, participate in type 2 immune responses to larger extracellular pathogens such as helminths [84]. Th17 cells, characterized by the expression of IL-17/IL-22 lineage cytokines and the master transcription factor RORγt, participate in type 3 immune responses to extracellular pathogens including some bacteria and fungi [85]. On the other hand, Treg cells regulate immune responses to maintain immune cell homeostasis and prevent immunopathology, rather than exerting effector functions like Th1/Th2/Th17 cells [86, 87]. The function of cytotoxic T cells (CD8+ T cells) is to directly kill pathogen-infected and tumor cells [83]. By recognizing and killing target cells, CD8+ T cells play an important role in immune clearance [88].

**Figure 5:**
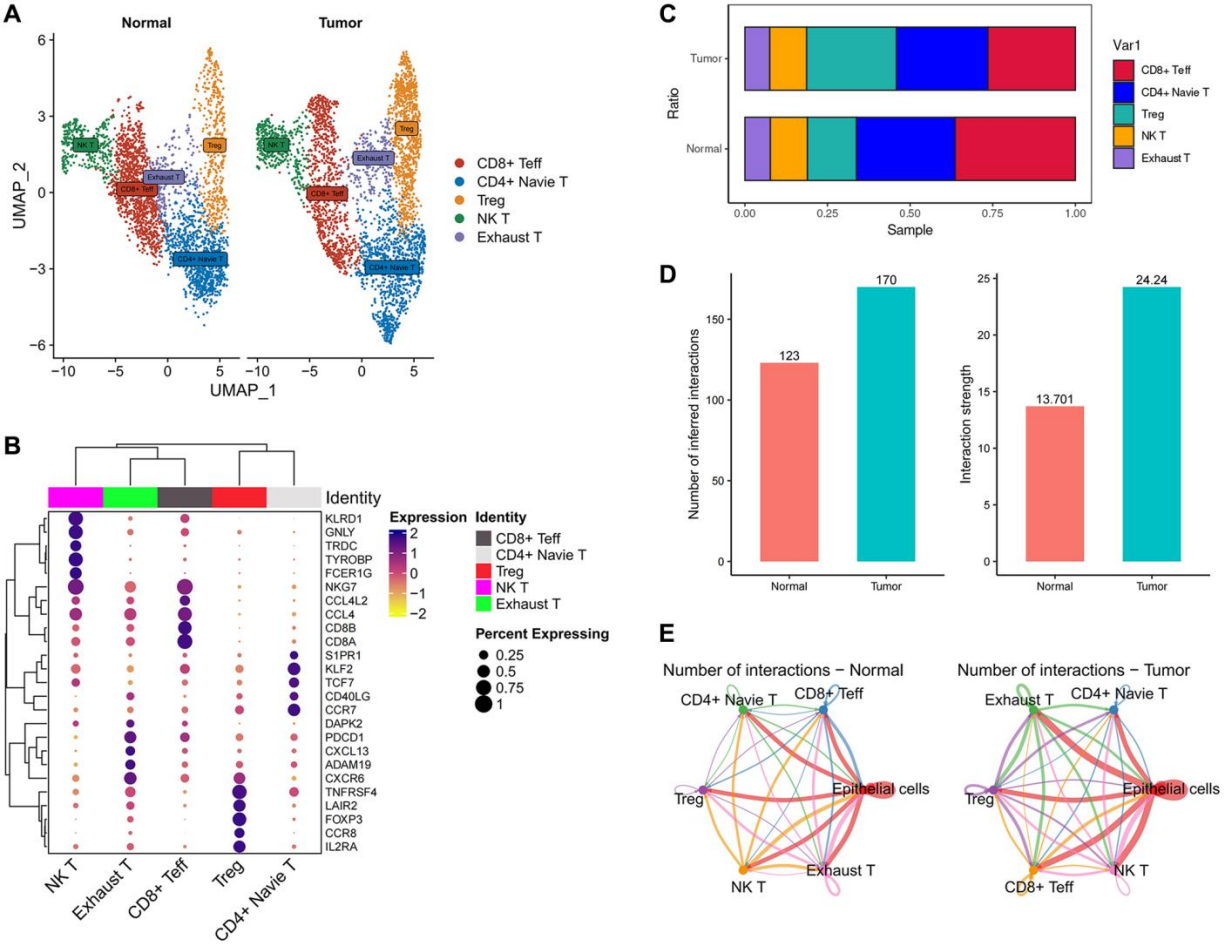
**The immune landscape of T cells in esophageal cancer.** (**A**) UMAP plot showing the subtypes of T cells. (**B**) Dotplot showing marker gene expression in indicated cell types. (**C**) Histograms indicating the proportion of T cells subtypes in tumor tissue and normal tissue. (**D**) Histograms indicating the cellular communication strength and quantity in tumor tissue and normal tissue. (**E**) Cell-Chat reveals possible cell-to-cell interactions.

To investigate the infiltration of T cells in the microenvironment of esophageal tumors, we undertook a further classification of T cells into five distinct subgroups using unsupervised clustering techniques (Figure 5A). Among these subgroups, CD8+ effector T cells (CD8+ Teff) displayed heightened expression of NKG7, CD8A, and CD8B; NK T cells exhibited increased expression of KLRD1, GNLY, and FCER1G; Exhausted T cells (Exhaust T) demonstrated elevated expression of PDCD1 (PD-1); T regulatory cells (Treg) displayed enhanced expression of FOXP3 and IL2RA; Naive T cells (Naive T) exhibited pronounced levels of TCF7 and CCR7. These findings align with previous literature reports (Figure 5B). We conducted an analysis of T cell infiltration by assessing the ratio of T cells, which revealed a substantial augmentation in Treg infiltration and a notable reduction in CD8+ effector T cells (CD8+ Teff) within tumor tissues, in comparison to normal tissues (Figure 5C).

Regulatory T cells (Tregs) are categorized into two distinct types: natural/thymic Tregs and peripherally induced Tregs, depending on their site of development. FOXP3+ natural Tregs are generated within the thymus and represent a functionally mature subset of T cells that are specialized in immune suppression (referred to as natural/thymic Tregs) [89–91]. The infiltration of Treg cells within tumors is closely associated with tumor development and prognosis. High levels of Treg cell infiltration have been correlated with tumor growth, invasion, and metastasis [92–94]. Studies have indicated that alterations in the quantity and functionality of Treg cells can result in immune tolerance, thereby enhancing the ability of tumor cells to evade immune surveillance [95, 96]. Consequently, the inhibition or modulation of Treg cell function has become an important strategy in cancer immunotherapy [97]. Effector T cells play a crucial role in tumor immunity. Specifically, effector T cells first recognize tumor-specific antigens (which can be proteins abnormally expressed by tumor cells, neoantigens generated by mutations, or overexpressed antigens) and subsequently become activated to initiate their cytotoxic mode [98]. Activated effector T cells undergo proliferation, differentiation, and functional regulation, releasing cytotoxic agents and pro-inflammatory cytokines that directly induce tumor cell apoptosis [99–101]. However, tumor cells can employ various mechanisms to evade and resist the attacks of effector T cells. They can reduce the expression of tumor antigens, produce immune inhibitory molecules (such as PD-L1) [102], and recruit immunosuppressive cells (such as Treg cells) [92–94], thereby attenuating the cytotoxic capabilities of effector T cells.

Notably, we did not detect a substantial difference in the infiltration of exhausted T cells (Exhaust T) between the two groups. This observation indicates that the modification in T cell composition within tumor tissues predominantly entails a specific reconfiguration in the equilibrium between Treg and CD8+ effector T cells, while exhausted T cells exhibit relatively minimal variation (Figure 5C). The principle of PD-L1 therapy for cancer involves the utilization of anti-PD-L1 antibodies or PD-1 antibodies to block the PD-1/PD-L1 signaling pathway. This blockade aims to alleviate the inhibitory effect of tumors on immune cells, restore immune cell activity, and enhance their ability to attack tumors [103, 104]. This observation indicates that the effectiveness of PD-1 may not be prominent in the case of esophageal tumors since there was no substantial alteration in the infiltration of exhausted T cells (Exhaust T), which are typically associated with PD-1 expression, between tumor and normal tissues. These findings suggest that other factors or mechanisms might exert greater influence on the immune response and tumor microenvironment in esophageal tumors. Further investigations are necessary to comprehensively comprehend the underlying factors that contribute to the immune response in this specific context.

### Cellular communication between T cells and FABP6+ tumor cells

To gain further insights into the cellular interactions between FABP6+ tumor cells and T cells, we employed CellChat, an analysis tool, to investigate the communication patterns between FABP6+ tumor cells and T cells, with normal epithelial cells paired with T cells serving as a control group. By utilizing CellChat, our aim was to uncover the specific signaling pathways and communication networks that play a role in the crosstalk between these cell populations. Comparing to normal tissues, we observed that in tumor tissues, both the number and intensity of cell communication events between different cell populations were higher (Figure 5D). This suggests that the tumor microenvironment fosters increased cellular communication, potentially indicating more complex and active intercellular signaling networks within the tumor tissue. Our analysis revealed that both normal epithelial cells and FABP6+ tumor cells within the tumor tissue exhibited cellular interactions with various subtypes of T cells. This indicates that there is cellular communication between these cell populations, regardless of whether they originate from normal tissue or FABP6+ tumor cells within the tumor (Figure 5E).

**Figure 6:**
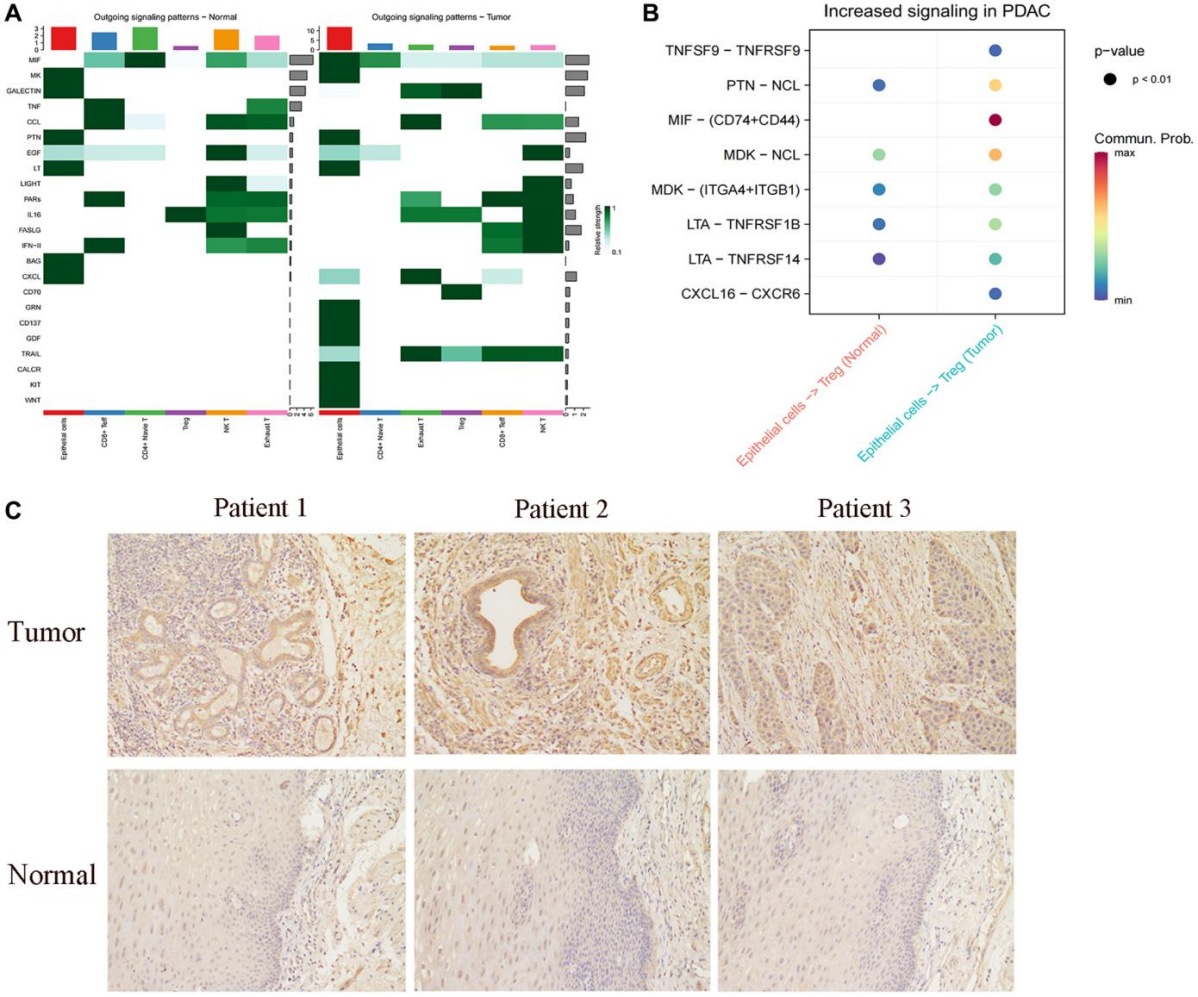
**Cellular communication between FABBP6+ esophageal cancer cells and normal epithelium and T cells.** (**A**) Heatmap showing the cellular communication signal output patterns. (**B**) Heatmap showing the ligand-receptor. (**C**) Immunohistochemistry staining statistics results for normal esophageal tissues and ESCA tissues. Scale bar: 50 μm. (^*^p < 0.05, ^**^p < 0.01, ^***^p < 0.001, ns indicates no statistical significance).

We proceeded to analyze the cellular communication output signal patterns of these cells. Interestingly, we observed a significant decrease in the CXCL-related pathway in FABP6+ tumor cells compared to normal tissues (Figure 6A). The CXCL family of cytokines plays a crucial role in tumor metastasis and invasion. Cytokines such as CXCL8, CXCL9, and CXCL10 are capable of attracting and activating immune cells, including macrophages and lymphocytes, causing their accumulation around the tumor. Through the release of substances that can dissolve tumor cells, these immune cells can limit the spread and dissemination of the tumor [105–107]. CXCL9 and CXCL10, by binding to their receptor CXCR, can attract and activate anti-tumor immune cells such as CD8+ T cells and natural killer (NK) cells, thereby enhancing their ability to eliminate tumor cells [108, 109]. Compared to FABP6+ tumor cells, normal tissues were found to lack the MIF-related pathway in our analysis of cellular communication output signal patterns. This suggests that the communication signals involving MIF may be absent or downregulated in normal tissues. The differential presence or absence of the MIF-related pathway between FABP6+ tumor cells and normal tissues highlights a potential role of MIF signaling in the tumorigenic processes associated with FABP6+ tumor cells. In order to further explore the specific receptors involved in the MIF pathway in FABP6+ tumor cells, we conducted an analysis and found a significant enrichment of CD74 and CD44 receptors in the tumor tissue (Figure 6B). MIF, CD74, and CD44 exhibit interplay and mutual regulation mechanisms in tumor development and progression. In addition to its expression in immune cells, CD74 is overexpressed in various tumor types and can form a complex with MIF, thereby modulating MIF’s function [110–112]. The interaction between MIF and CD74 can enhance the stability and biological activity of MIF, influencing tumor development and progression [113]. CD44, a cell surface molecule, is widely expressed in multiple cell types, including tumor cells. Elevated expression of CD44 in tumors is associated with characteristics of tumor stem cells, cell migration, and infiltration processes [114–117]. CD44 in tumors may interact with MIF and CD74, participating in the regulation of related signaling pathways [118–121]. Specifically, MIF can activate downstream signaling pathways such as MAPK (Mitogen-Activated Protein Kinase) and PI3K (Phosphoinositide 3-Kinase) by binding to CD74, promoting tumor cell proliferation, survival, and metastasis [113, 121]. Furthermore, the high expression of CD74 can also impact the expression and function of CD44. The interaction between CD74 and CD44 may influence tumor cell behavior through the regulation of cell-matrix interactions, cell adhesion, migration, and infiltration pathways [119, 120]. Existing studies have found that, TGF-β expressing B lymphocytes assembled into clusters and engaged with T cells through lymphocytic recruitment signals (SELL, CXCL13, CCL4, CD74), while interacting with regulatory T cells via CD47:SIRP-γ and FOXP3 promoting Galectin-9:CD44 pathways. This suggests that FABP6+ tumor cells may utilize CD74 to interact with CD44 on Treg cells, potentially promoting the immunosuppressive function of Treg cells and facilitating tumor development. This observation highlights the potential role of the CD74-CD44 interaction in modulating immune responses within the tumor microenvironment and provides insights into the mechanisms underlying immune evasion and tumor progression.

To further explore FABP6 expression in ESCA, we used IHC to detect FABP6 expression at the protein level. IHC staining for FABP6 was negative in normal esophageal specimens and positive in ESCA specimens (Figure 6C). Thus, we hypothesized that FABP6 is an unfavorable prognostic biomarker in ESCA cancer.

### The migratory potential of ESCA cells is observed to be inhibited upon knockdown of FABP6

**Figure 7:**
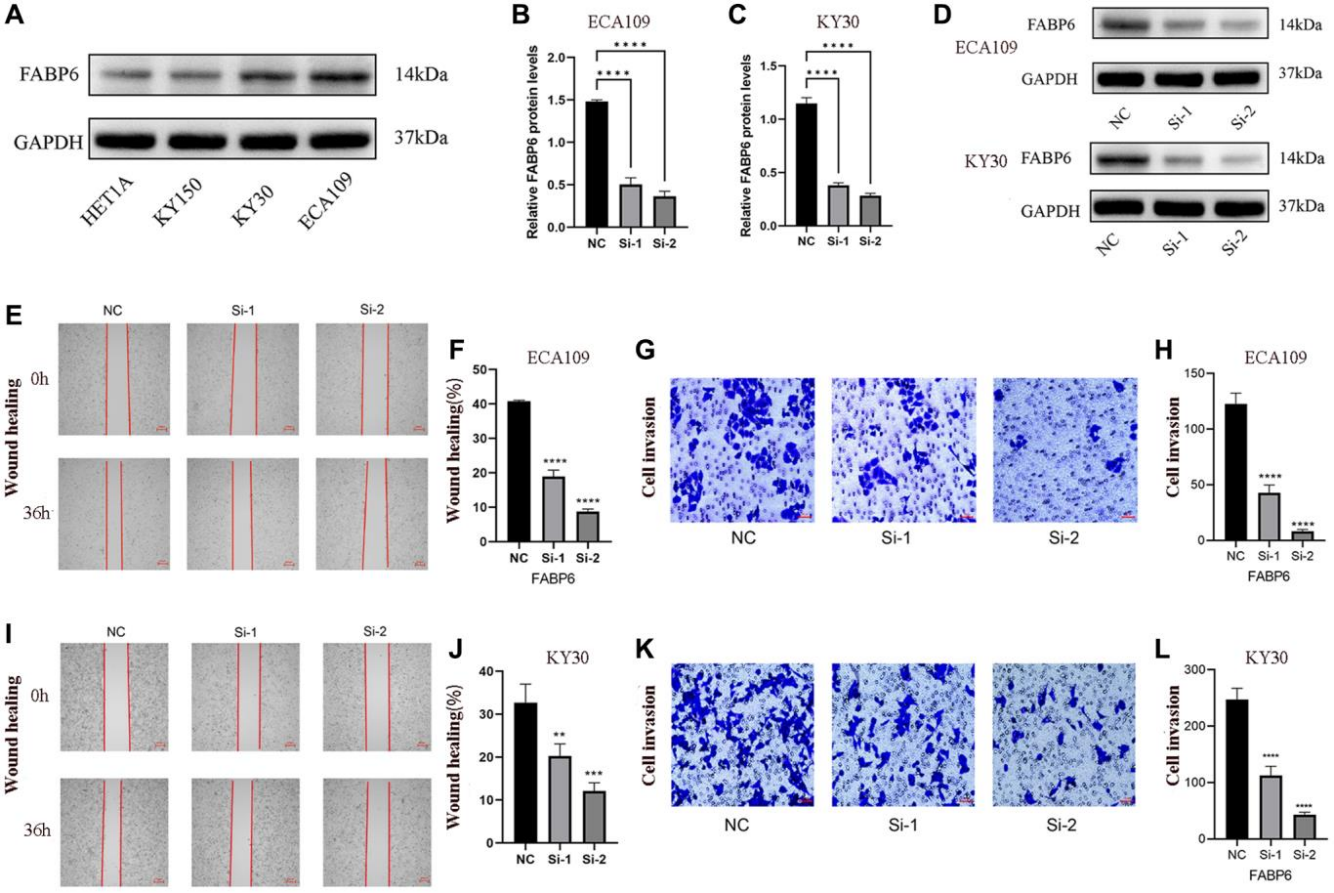
**Displays the experimental verification of our study.** (**A**) showcases the expression of FABP6 in diverse esophageal cancer cell lines, providing evidence of FABP6’s promotion of migratory capability in ESCA cells. (**B**–**D**) present the results obtained from qPCR and Western blotting analyses, which demonstrated the successful silencing of FABP6 expression in the ECA-109 and KYSE30 cell lines using siRNA. For the ECA109 cell line, Scratch wound-healing assays (**E**, **F**) and transwell migration healing assays (**G**, **H**) were conducted to evaluate the migration capability influenced by FABP6, accompanied by their respective statistical representations. Similarly, for the KYSE30 cell line, Scratch wound-healing assays (**I**, **J**) and transwell migration healing assays (**K**, **L**) were performed to assess migration ability regulated by FABP6, along with corresponding statistical illustrations. The scale bar for Figure 7E and 7I represent 250 μm, while Figure 7G, 7K feature a scale bar of 50 μm. Statistical analysis revealed significant differences between the groups (^*^p < 0.05, ^***^p < 0.001).

To elucidate the role of FABP6 in ESCA, we initially selected ECA109 and KYSE30 cell lines based on western blotting tests to establish si-FABP6 cell lines (Figure 7A). Subsequently, we quantified FABP6 protein levels using RT-PCR and protein blotting techniques (Figure 7B–7D). Furthermore, we conducted wound-healing and transwell assays to investigate the impact of FABP6 expression on cell migration and invasion in ESCA (Figure 7E, 7G, 7I, 7K). Our results demonstrated that FABP6 knockdown significantly reduced the migration potential of ECA109 and KYSE30 cell lines (Figure 7F, 7H, 7J, 7L). In conclusion, our findings suggest that FABP6 may expedite the development of ESCA by augmenting the migratory potential of cells.

### Visualization of co-expression of FABP6 and FOXP3

**Figure 8:**
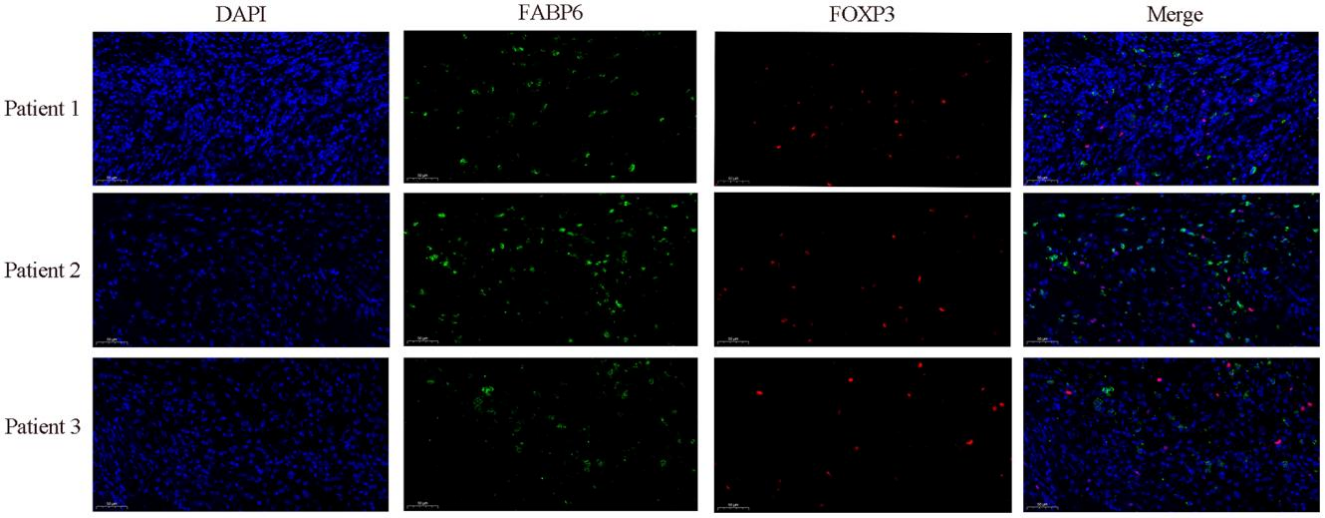
**Immunofluorescence staining further verified the relationship between FABP6 expression and Treg infiltration.** Immunofluorescence staining was performed on esophageal cancer specimens to examine FABP6 expression and Treg infiltration. The Treg lineage-specifying transcription factor FOXP3 served as a Treg marker. FABP6 (green), FOXP3 (red) and DAPI (blue) staining. Scale bar, 50 μm.

In our preceding investigation, we conducted exploratory analysis utilizing single-cell data from esophageal carcinoma, revealing a regulatory influence of FABP6 on Treg cells, demonstrating a positive correlation. To validate this regulatory relationship, we employed immunofluorescence techniques to ascertain the expression dynamics between FABP6 and the marker gene FOXP3 in Treg cells. The observed upregulation of FABP6 was associated with a notable enhancement in Treg cell proliferation, concurrently promoting heightened expression levels of FOXP3 (Figure 8).

## DISCUSSION

The results of the study provide valuable insights into the tumor microenvironment in primary esophageal tumors, specifically focusing on the characterization of cell types, the heterogeneity of esophageal tumor cells, the role of FABP6+ tumor cells, the infiltration of T cells, and the cellular communication between T cells and FABP6+ tumor cells.

Firstly, the scRNA-seq analysis identified distinct clusters of cells within the tumor microenvironment, including non-immune cells such as fibroblasts, myofibroblasts, endothelial cells, and epithelial cells, as well as immunocytes such as myeloid-derived cells, T cells, B cells, and plasma cells. The observation of substantial heterogeneity in the composition of cell types in normal and tumor tissues suggests the importance of non-immune cells in tumor growth and development. Moreover, the infiltration of immune cells revealed an increase in myeloid cells and a decrease in plasma cells within tumor tissues, indicating potential immune cell dysregulation in the tumor microenvironment.

The heterogeneity of esophageal tumor cells was explored through copyKAT analysis, which identified a distinct subset of tumor cells referred to as cluster 0. This cluster exhibited higher chromosomal variation, suggesting a polyploid nature of esophageal tumor cells. The analysis further revealed a developmental lineage of tumor cells, with cluster 0 being positioned as the starting point of cell differentiation. Notably, cluster 0 showed higher expression of genes associated with fatty acid metabolism, indicating its potential role in energy generation for proliferation and development. This finding is supported by the literature, which suggests that reprogramming fatty acid metabolism can promote tumor growth.

FABP6, identified as a defining marker for cluster 0, was found to be highly expressed in these tumor cells compared to normal epithelial cells. The analysis of the TCGA database confirmed higher expression of FABP6 in esophageal tumor tissue, and patients with high FABP6 expression exhibited poorer survival. The enrichment of transcription factors MYC and TAF7 in cluster 0, along with pathway enrichment analysis, revealed the activation of the MAPK pathway in these FABP6+ tumor cells. These findings suggest the potential involvement of FABP6 in regulating fatty acid metabolism and signaling pathways related to tumor progression. Regarding T cell infiltration in esophageal tumors, the analysis identified distinct subsets of T cells, including CD8+ effector T cells, NK T cells, exhausted T cells, T regulatory cells (Tregs), and naive T cells. Comparing tumor and normal tissues, the infiltration of Tregs was significantly increased, while CD8+ effector T cells showed a significant decrease in tumor tissues. Interestingly, no significant change was observed in exhausted T cell infiltration, suggesting that the efficacy of PD-1 may not be significant in esophageal tumors. This indicates that other factors or mechanisms may play a more influential role in the immune response and tumor microenvironment of esophageal tumors.

The study also investigated the cellular communication between FABP6+ tumor cells and T cells. The analysis revealed increased communication events within tumor tissues, indicating a more complex intercellular signaling network in the tumor microenvironment. Both normal epithelial cells and FABP6+ tumor cells exhibited cellular interactions with various T cell subtypes, highlighting the presence of communication between these cell populations. Differential signaling pathways, such as CXCL and MIF-related pathways, were observed between FABP6+ tumor cells and normal tissues. The enrichment of CD74 and CD44 receptors in the tumor tissue suggests the potential interaction between FABP6+ tumor cells and Treg cells, which may contribute to immunosuppression and tumor development. Some limitations exist, including the need for functional validation of the bioinformatics predictions. Future directions entail experimentally confirming the role of FABP6 in potentiating tumor growth, metastasis, and Treg recruitment. Animal models can elucidate the *in vivo* impact of targeting FABP6. Broader patient cohorts are required to validate FABP6 as a prognostic biomarker. Overall, this single-cell sequencing study sheds light on tumor heterogeneity and identifies FABP6 as a potential driver of esophageal carcinoma progression and immunotherapy resistance, meriting further investigation as both a predictive biomarker and therapeutic target.

## CONCLUSION

This study provides novel insights into the tumor microenvironment and heterogeneity of esophageal squamous cell carcinoma through comprehensive single-cell RNA sequencing analysis. Key findings demonstrate an enrichment of FABP6-expressing tumor cells with high fatty acid metabolism, alterations in T cell infiltration patterns, and increased cellular communication between FABP6+ tumor cells and Tregs. The characterization of tumor cell subpopulations reveals a precursor cluster with high FABP6 expression, MAPK pathway activation, and poor patient survival.
